# Comparison of Cognitive Intervention Delivery Formats in Patients with Dementia-A Network Meta-analysis

**DOI:** 10.1093/geroni/igab046.2456

**Published:** 2021-12-17

**Authors:** Yue Sun, Zhi-wen Wang

**Affiliations:** 1 School of Nursing, School of Nursing, Peking University, Beijing, China (People's Republic); 2 Peking University Health Science Center, Beijing, Beijing, China (People's Republic)

## Abstract

Cognitive intervention has been shown to be effective to delay cognitive decline in older adults with dementia. However, whether cognitive intervention could be effectively delivered in individual, group, telephone, guided self-help and unguided self-help formats remains unclear. Pubmed, Embase, Cumulative Index to Nursing and Allied Health Literature (CINAHL), CINAHL, the Cochrane Central Register of Controlled Trials, Web of science, China National Knowledge Infrastructure database, Chinese Biomedical Literature database and Wan Fang database were systematically searched. 3419 records were extracted, quality assessed, and double-blind screened by 2 authors. Totally 51 studies were included which enrolled 3388 participants. Network meta-analysis (NMA) was conducted to evaluate the relative effects and rank probability of different cognitive intervention delivery formats. For older adults with dementia, guided self-help, group and individual cognitive intervention delivery formats appeared effective in improving the cognitive function, while telephone and unguided self-help were not significantly inferior to control condition. Guided self-help had the highest probability of being the best treatment among the five cognitive intervention delivery formats. Health-care professionals should apply personalized cognitive intervention format based on individual condition and preferences.

